# Effects of Early Frequent Nephrology Care on Emergency Department Visits among Patients with End-stage Renal Disease

**DOI:** 10.3390/ijerph16071158

**Published:** 2019-03-31

**Authors:** Yun-Yi Chen, Likwang Chen, Jenq-Wen Huang, Ju-Yeh Yang

**Affiliations:** 1 Institute of Health Policy and Management, College of Public Health, National Taiwan University, Taipei 100, Taiwan; yunyichen@gmail.com; 2 Institute of Population Health Sciences, National Health Research Institutes, Zhunan 350, Taiwan; likwang@nhri.org.tw; 3 Division of Nephrology, Department of Internal Medicine, National Taiwan University Hospital, Taipei 100, Taiwan; jenqwen@gmail.com; 4 Division of Nephrology, Far Eastern Memorial Hospital, New Taipei City 220, Taiwan; 5 Department of Quality Management Center, Far Eastern Memorial Hospital, New Taipei City 220, Taiwan; 6 Lee-Ming Institute of Technology, New Taipei City 243, Taiwan

**Keywords:** end-stage renal disease (ESRD), chronic kidney disease (CKD), dialysis initiation, quality of care, predialysis nephrology care, early referral, emergency department visits, infection, avoidable emergency department visits

## Abstract

In this retrospective cohort study, we examined the association between predialysis nephrology care status and emergency department (ED) events among patients with end-stage renal disease. Data pertaining to 76,702 patients who began dialysis treatment between 1999 and 2010 were obtained from the National Health Insurance Research Database of Taiwan (NHIRD). The patients were divided into three groups based on the timing of the first nephrology care visit prior to the initiation of maintenance dialysis, and the frequency of nephrologist visits (i.e., early referral/frequent consultation, early referral/infrequent consultation, late referral). At 1-year post-dialysis initiation, a large number of the patients had experienced at least one all-cause ED visit (58%), infection-related ED visit (17%), or potentially avoidable ED visit (7%). Cox proportional hazard models revealed that patients who received early frequent care faced an 8% lower risk of all-cause ED visit (HR: 0.92; 95% CI: 0.90–0.94), a 24% lower risk of infection-related ED visit (HR: 0.76; 95% CI: 0.73–0.79), and a 24% lower risk of avoidable ED visit (HR: 0.76; 95% CI: 0.71–0.81), compared with patients in the late referral group. With regard to the patients undergoing early infrequent consultations, the only marginally significant association was for infection-related ED visits. Recurrent event analysis revealed generally consistent results. Overall, these findings indicate that continuous nephrology care from early in the predialysis period could reduce the risk of ED utilization in the first year of dialysis treatment.

## 1. Introduction

Researchers around the globe have noted increases in the number of people diagnosed with end-stage renal disease (ESRD) and the number of patients receiving maintenance dialysis treatment [1,2]. Compared to the general population, ESRD patients are generally older individuals with inferior health and multiple comorbidities. As a result, ESRD is associated with high morbidity levels and a high risk of early death [3]. ESRD also imposes high social costs and is a considerable draw on medical resources. A number of researchers have recommended that patients with end-stage of chronic kidney disease (CKD) be referred to specialist nephrology services as soon as possible in order to prepare for long-term dialysis and manage the various risk factors associated with this disease [4]. Early nephrology referral is particularly important for patients with advanced CKD.

Early referral can help to slow the progression to ESRD and diminished renal function [5,6]. It has also been associated with reduced healthcare costs [7], reduced risk of cardiovascular events [8], reduced risk of early technique failure [9], and lower hospitalization and mortality rates [5,10,11,12,13,14]. Patients who receive an early referral tend to enjoy more quality-adjusted life-years (QALYs) [15], superior placement of dialysis access [12,16], and greater freedom in the selection of dialysis modality [17]. However, the association between patterns in predialysis care and visits to the emergency department (ED) has yet to be elucidated.

Patients undergoing dialysis tend to make frequent visits to the emergency department (ED) [18,19], particularly those receiving maintenance hemodialysis [20]. Even after controlling for age and sex, these patients are eight times more likely than those not receiving dialysis to be transferred to an ED [21]. This is a clear indication of the need for strategies and interventions to reduce the health risks facing dialysis patients. One previous study included nephrology care as a prime factor affecting ED use within one year after the initiation of dialysis in patients with ESRD [22]; however, there was no mention of the timing with which nephrology care was implemented.

One recent study reported that the timing of referrals and the frequency of nephrology care strongly influence the healthcare received by patients approaching the ESRD stage [8]. In this study, we employed long-term data in characterizing the association between the timely implementation of continuous predialysis nephrology care and the risk of ED visits within the first year after beginning long-term dialysis. This study was based on the hypothesis that early frequent nephrology care reduces the probability of all-cause, infection-related, and potentially avoidable ED visits among patients diagnosed with ESRD.

## 2. Methods 

### 2.1. Dataset

The data used in this study was obtained from the National Health Insurance Research Database (NHIRD) of Taiwan, which is maintained by the National Health Research Institute (NHRI). We obtained a random sample of patients who had either received dialysis care, or had a principal diagnosis of acute kidney injury, CKD, or a severe neurological disease during the study period (1997–2011). The random sample included two million patients (random sampling fraction = 71%). The research data included demographic information, diagnoses, treatment procedures, prescriptions, health insurance information, and the type of medical service providers (for outpatients and inpatients). To ensure patient confidentiality, the database includes only de-identified information, and linkages to other databases are prohibited. This study was approved by the institutional review board of the Far Eastern Memorial Hospital (approval number 102167-E), which waived the requirement for informed consent.

### 2.2. Design and Study Participants

This observational retrospective cohort study enrolled ESRD patients who started receiving long-term dialysis therapy between 1 January 1999 and 31 December 2010. Long-term dialysis was defined as dialysis treatment spanning >90 days, during which the interval between each session was <60 days. We excluded patients with missing information related to date of birth or sex as well as patients aged <20 years at the time of the first dialysis treatment.

Patient tracking was conducted from the initiation of long-term dialysis to the occurrence of an ED event, the receipt of a successful kidney transplant, death, loss to follow-up, or the end of the study period (365 days post-dialysis). The dates of deaths were confirmed by referring to the registry of catastrophic illness or the patient’s discharge status. In cases where the medical care records of a patient were not updated for more than one year, the patient was deemed lost to follow-up.

### 2.3. Variables

#### 2.3.1. Independent Variables

We adopted the approach outlined by Yang et al. [8] to divide the patients into three groups based on predialysis care patterns (i.e., the time of referral to a specialist and the frequency of consultations with a nephrologist). The groups are described as follows: (1) late nephrology care group: patients who made their first visit to a nephrologist within 6 months prior to the initiation of long-term dialysis; (2) early frequent nephrology care group: patients who made their first visit to a nephrologist more than six months prior to the initiation of long-term dialysis and made at least one visit to a nephrologist every three months; (3) early infrequent nephrology care: patients who made their first visit to a nephrologist more than six months prior to the initiation of long-term dialysis and made visits to a nephrologist at intervals exceeding three months.

#### 2.3.2. Outcome Variables

Analysis was performed to identify all-cause ED visits, infection-related ED visits, and potentially avoidable ED visits within the first year following the initiation of dialysis. ED events were coded using discharge diagnosis codes from the international classification of disease, ninth revision, clinical modification (ICD-9-CM).

Infection-related ED use included ED visits for bacteremia, endocarditis, peritonitis, septicemia, gastrointestinal infection, genitourinary infection, joint or bone infection, pulmonary infection, and soft-tissue infection [23]. The definition and technical specifications of potentially avoidable ED use were obtained from the prevention quality indicators (PQIs) version 6.0 prepared by the agency for healthcare research and quality (AHRQ) [24]. 

We adopted the AHRQ definitions for overall, acute, and chronic composite PQIs in assessing potentially avoidable ED visits and ED visits related to acute disease and chronic disease conditions. Each composite indicator was aggregated at the patient level. The criteria used to define a potentially avoidable ED visit included the occurrence of any of the following PQIs: bacterial pneumonia, urinary tract infection, dehydration, adult asthma, chronic obstructive pulmonary disease, short- or long-term complications from diabetes mellitus, uncontrolled diabetes mellitus, diabetes mellitus–related lower extremity amputation, hypertension, and heart failure. The criteria used to define acute disease condition included ED visits for dehydration, bacterial pneumonia, urinary tract infection, and dehydration. The criteria used to define chronic disease condition included ED visits for adult asthma, chronic obstructive pulmonary disease, short or long-term complications from diabetes mellitus, uncontrolled diabetes mellitus, diabetes mellitus–related lower extremity amputation, hypertension, and heart failure.

### 2.4. Covariates

The covariates in this study included the year in which long-term dialysis was initiated and the age, sex, economic status, dialysis modality, and baseline health status of the patients. The baseline health status of the patients was estimated using the occurrence of hospitalization, the number of physician visits, Deyo’s Charlson comorbidity index (CCI), and comorbid conditions within one year prior to the initiation of long-term dialysis.

The NHIRD does not list socioeconomic indicators (e.g., education or personal/household income); therefore, we sought to establish the economic status of the patients based on the level of their insurance premiums. Dialysis modality refers to methods established within three months after the initiation of maintenance dialysis treatment. Outpatient clinic visits were divided into three equal tertiles. CCI and comorbidities were calculated in cases where a diagnosis had been recorded at least three times in the outpatient claim records or at least one time in the inpatient claim records. Comorbid diseases included arrhythmia, cancer, cerebral vascular disease, chronic liver disease, chronic obstructive pulmonary disease, coronary artery disease, dementia, diabetes, dyslipidemia, gout, heart failure, hypertension, osteoporosis, peripheral vascular disease, peptic ulcer disease, psychiatric disorders, and valvular heart disease.

### 2.5. Statistical Analysis

Prior to conducting the analysis, the distributions of the continuous variables were assessed using the Kolmogorov-Smirnov test. In cases where the data were normally distributed, analysis of variance was used for continuous variables; otherwise, if the data were non-normally distributed, the Kruskal-Wallis test was used to compare between-group differences. The chi-square test was used for categorical variables. The Cox proportional hazard model was used to evaluate the association between patterns in predialysis care and the first ED event in the early dialysis period. Considering that participants could have repeated ED events during follow-up, we employed proportional rates and proportional means regression models for recurrence data [25].

Sensitivity analysis was conducted to determine the composite outcome of an ED event or death. Subgroup analysis was performed according to age, sex, economic status, treatment modality, CCI, and diabetes status to evaluate potential effect modifiers. All analyses were performed using SAS software version 9.4 (SAS Institute Inc., Cary, NC, USA). 

## 3. Results

### 3.1. Patient Characteristics and Nephrology Care Status

**Figure 1:**
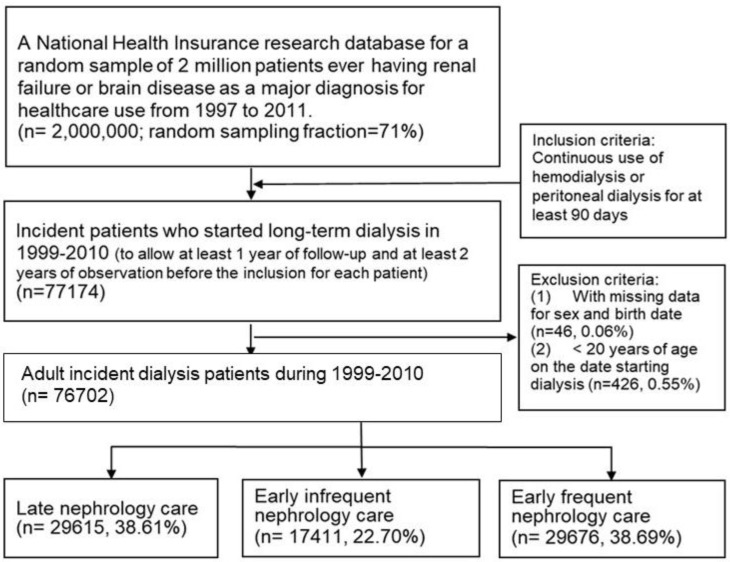
Flow chart of cohort establishment.

Figure 1 presents a flowchart showing the patient recruitment process. Between 1999 and 2010, a total of 77,174 patients in the database started long-term dialysis; however, 426 patients were excluded due to age and 46 were excluded due to missing data. Among the remaining 76,702 eligible patients, 38.7% received early frequent care, 22.7% received early infrequent care, and 38.6% received late nephrology care.

Compared with the other groups, the early frequent group included patients with a higher average age and higher economic status. Furthermore, most of the patients in the early frequent group were women and underwent peritoneal dialysis. Within one year prior to the initiation of long-term dialysis, patients in the early frequent group were more likely to visit outpatient clinics; however, they were less likely to be admitted to hospital. Patients in the early infrequent care group were more likely to develop comorbid conditions. Baseline characteristics in predialysis referral patterns are listed in Table 1. Baseline characteristics of patients with and without ED visit are shown in Appendix A, Table A1.

### 3.2. ED Event Distribution

During follow-up, a large number of the patients experienced at least one all-cause ED visit (58%), infection-related ED visit (17%), or potentially avoidable ED visit (7%). The mean number of ED visits per patient-year were as follows: all-cause ED visits (1.73), infection-related ED visits (0.28), and potentially avoidable ED visits (0.09). Patients who received early referrals and made frequent visits to their physician had the lowest mean number of ED visits, and had a lower rate of frequent ED users (i.e., equal to or more than three times visits to the ED) (Table 2).

### 3.3. Risk of ED Events within 1-Year after Beginning Dialysis

Compared to patients in the late group, the adjusted hazard ratio (HR) of all-cause ED visits was 0.92 for patients receiving early frequent care (95% confidence interval [CI]: 0.90–0.94) and 1.01 for patients receiving early infrequent care (95% CI: 0.99–1.04) (Table 3).

After adjustment for covariates, patients receiving early frequent care faced a 24% lower risk of infection-related ED visit (HR: 0.76; 95% CI: 0.73–0.79), whereas patients receiving early infrequent care experienced 6% lower risk of infection-related ED visit (HR: 0.94; 95% CI: 0.90–0.98).

In terms of potentially avoidable ED visits, patients in the early frequent group faced a 24% lower risk for all-condition avoidable ED visits (HR: 0.76; 95% CI: 0.71–0.81) compared to patients in the late group. The effects of early frequent care on acute-condition and chronic-condition avoidable ED visits followed the same trend (data not shown).

**Figure 2:**
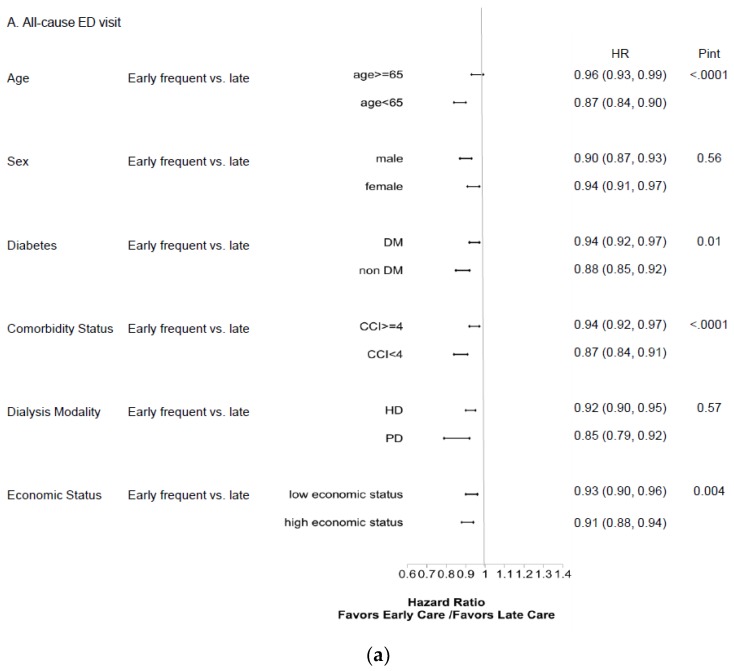

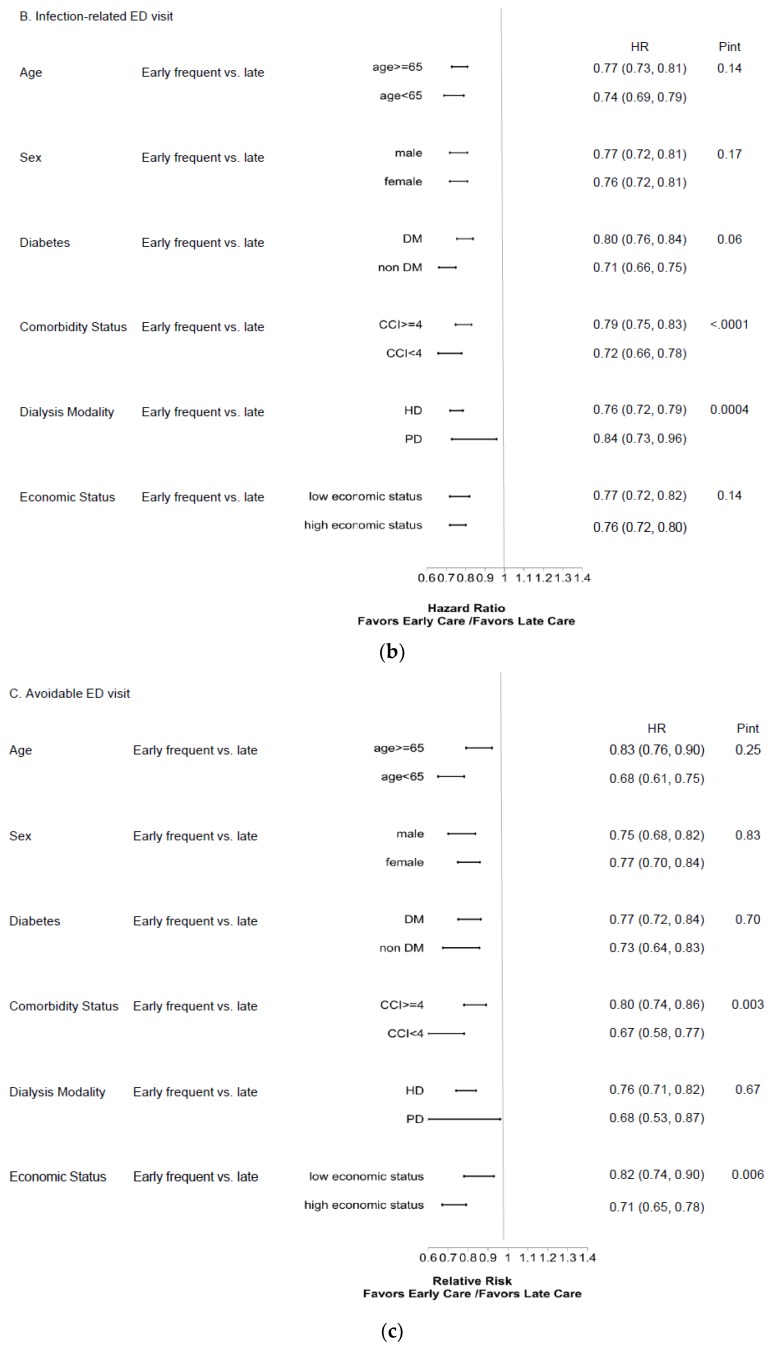
Subgroup analysis of hazard ratio for 1-year ED visit between the early-frequent and late-nephrology-care groups for first event of (**a**) all-cause, (**b**) infection-related, and (**c**) potentially avoidable ED visit. Abbreviations: ED, emergency department; CCI, Deyo’s Charlson Comorbidity Index; DM, diabetes mellitus; HD, hemodialysis; HR, hazard ratio; PD, peritoneal dialysis; Pint, *p* value for interaction term.

**Figure 3:**
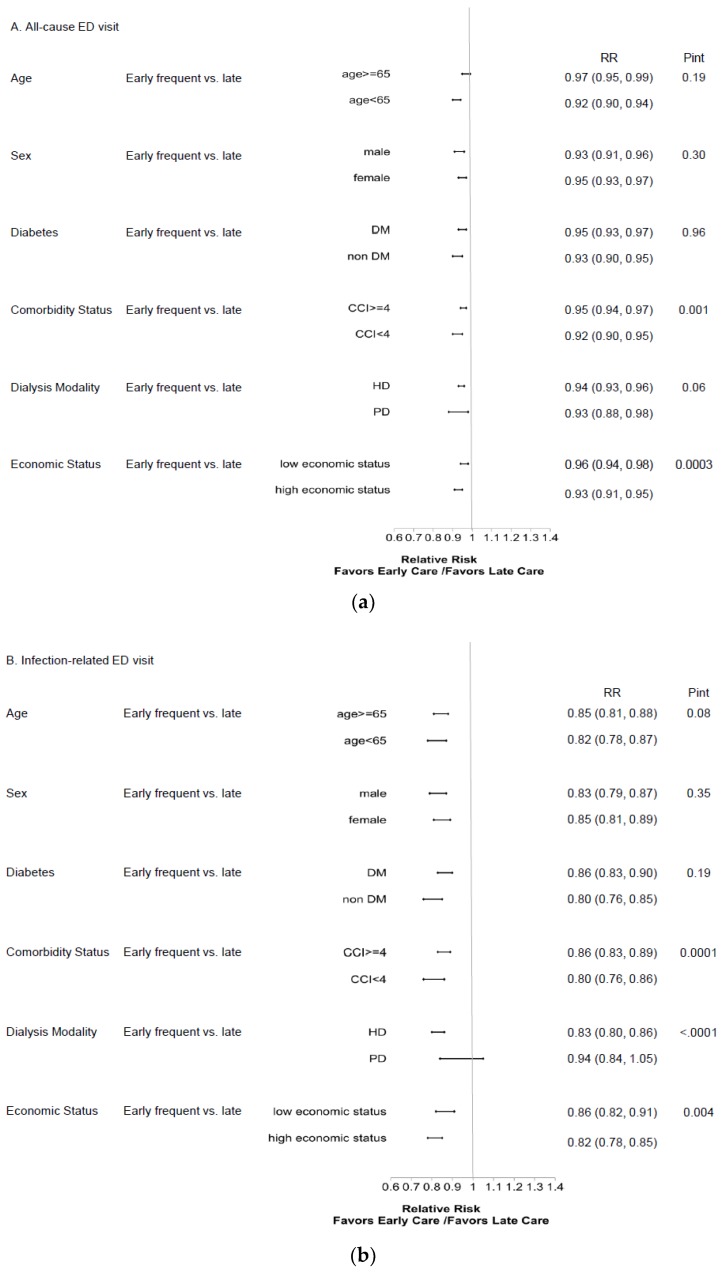

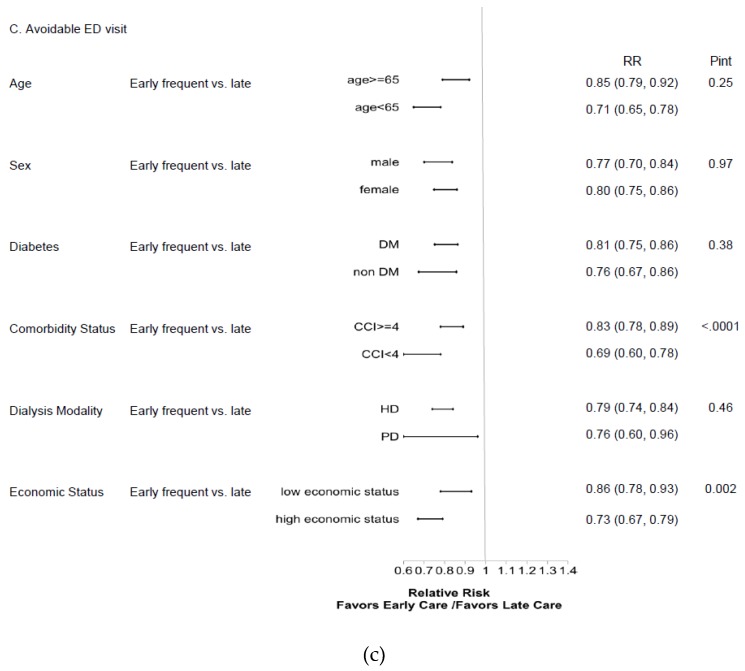
Subgroup analysis of relative risk for 1-year ED visits between the early-frequent and late-nephrology-care groups for recurrent events of (**a**) all-cause, (**b**) infection-related, and (**c**) potentially avoidable ED visits. Abbreviations: ED, emergency department; CCI, Deyo’s Charlson Comorbidity Index; DM, diabetes mellitus; HD, hemodialysis; HR, hazard ratio; PD, peritoneal dialysis; Pint, *p* value for interaction term.

As shown in Table 3, our analysis of recurrent ED visits yielded results similar to those above. When death was considered a composite outcome in sensitivity analysis, the influence of early frequent care on first ED events anda recurrent ED events was consistent. Subgroup analysis revealed that younger patients, non-diabetics, patients with lower CCI scores, and those with higher economic status benefited the most from early frequent nephrology care in terms of improved healthcare outcomes (Figure 2 and Figure 3).

## 4. Discussion

In this study, we investigated the relationship between the timing and continuity of predialysis nephrology care and ED events among patients undergoing maintenance dialysis. This observational cohort study was based on data obtained from a comprehensive nationwide database (NHIRD) of healthcare utilization in Taiwan. Our results revealed that frequent nephrology care for ESRD patients in the early predialysis stage was associated with a lower risk of all-cause, infection-related, and potentially avoidable ED visits within the first year of dialysis (adjusted HR = 0.92, 0.76, and 0.76, respectively). Note that the time of care was not the sole issue. Early but infrequent care was associated with only a marginally lower risk of infection-related ED visit.

We discovered that 58% of the patients in this study were admitted to the ED within one year after beginning dialysis treatment, and the mean number of all-cause ED visits was 1.73 per patient-year during the study period (1999–2011). Two recent population-based studies investigated the incidence of ED visits among patients with ESRD. One U.S. Medicare cohort study reported that after initiating dialysis, 55% of the patients were admitted to ED within one year (2.89 ED visits per patient-year) [22]. Another Canadian cohort study reported 1.5 ED visits per patient-year [21]. It is likely that the high rate of ED visits among dialysis patients can be attributed to urgent or critical clinical situations, such as complications of vascular access or adverse cardiovascular events.

Researchers have previously reported that patients who received early referrals have better control over clinical parameters (e.g., blood pressure and serum levels of albumin, bicarbonate, calcium, cholesterol, hemoglobin, potassium, and phosphate), compared to patients who received referrals after an extended duration [11,13,26]. This is an indication that early referrals make it easier to manage health status and in so doing reduce the likelihood of escalation to urgent status. In this study, we discovered that patients who received referrals early and made frequent visits to their physician were less likely to be hospitalized within one year prior to initiating dialysis, despite the fact that patients in this group tended to be older and presented higher CCI scores. This means that frequent consultations with nephrologists before the disease has a chance to progress enhances the likelihood of a favorable outcome.

Nonetheless, it is important to note that the patient (not the physician) decides whether to visit the ED. This appears to indicate that early nephrological intervention may influence the self-management ability of the patient by enabling more informed decisions. The education of patients in terms of self-care could help them to identify signs of impending clinical difficulties and improve their ability to deal appropriately with important symptoms that might otherwise result in an ED visit. For instance, nephrologists could educate their patients of the dangers of swelling and/or rapid gain weight. Patients should be made aware that under these conditions, they should control their intake of dietary phosphorus, undergo dialysis earlier than scheduled, or perhaps take a diuretic instead of heading directly to the ED. Prior to dialysis, it is important that nephrologists enable the timely preparation of dialysis access in hemodialysis patients [27] in order to reduce the risk of systemic infection [28,29]. Many CKD patients with impaired kidney function take medication at inappropriate dosages [30,31]. Researchers have shown that adverse events associated with the inappropriate use of medications can increase the frequency of ED visits among older adults [32]. Nephrologists are more likely than primary care physicians to focus on appropriate dosing for patients with renal disease [33].

In terms of the initial contact with a nephrologist, the cutoff point between a late referral and early referral has been set at one month [27], three months [9,10,11,13], four months [14,17,34], six months [5,8,35,36], or one year [7,26,37]. Notwithstanding the variations in defining nephrology referral timing, Quaglia et al. mentioned that, “Predialysis nephrology care is a much wider concept than providing the patient with a dialysis access—a crucial but minimal requirement—and consequently demands a longer time (i.e., several years) to produce results.” [38]. In this study, we discovered that a referral six months prior to dialysis does not necessarily reduce the risk of unfavorable outcomes; however, an early referral in conjunction with frequent nephrologist consultations can reduce the risk of ED visits among patients with ESRD. The continuity of nephrology care makes a difference. With continuous care, nephrologists may coordinate the care plan and provide proactive care according to the spectrum of problems encountered in pre-ESRD patients. On the other hand, patients would have more chances to learn how to cope with the health challenges at predialysis stage with the continuous help and support from professionals.

This study has a number of limitations. First, the NHIRD does not list marital status, education, health literacy, family history, health behaviors, or social support. Moreover, we were unable to control for clinical values, such as the results of laboratory tests. Second, our results are susceptible to various forms of bias due to our inability to measure the rate of CKD progression to ESRD. Third, we were unable to determine the triage status of each ED stay, which made it impossible to exclude inappropriate (i.e., nonurgent) ED visits. Additionally, we considered only the timing and frequency of nephrology care prior to maintenance dialysis. We did not explore patient–provider relationships, health education content during the care process, the circulation and/or discussion of medical information between patients and care providers, or medication adherence. It is very likely that these factors could affect the outcomes of nephrology care. Otherwise, a retrospective observational design can only describe associations; i.e., it cannot be used to indicate causation. Further studies are required to validate actual causal relationships. Finally, predialysis management by primary care providers can vary considerably among regions [11]; thus, the generalizability of the study findings should be carefully verified.

To the best of our knowledge, this was the first study to use nationwide data to explore the association between continuity of predialysis nephrology care and ED events among patients with ESRD in the early dialysis period. The need of continuity of nephrology care in the pre-ESRD stage should be addressed. A suitable disease management regime can reduce health risks, improve health care quality for patients undergoing dialysis, and facilitate the efficient use of medical resources. We recommend that health policy makers and health professionals encourage patients approaching the end stage of CKD to seek consultation with a nephrologist in a timely manner, and educate patients concerning the continuity of nephrology care.

## 5. Conclusions

This study provides further evidence to support the contention that patients undergoing dialysis face a high risk of ED visits, and that timely and frequent consultations with a nephrologist during the predialysis stage is associated with a lower incidence of ED events within the first year of beginning dialysis.

## Figures and Tables

**Table 1:** Baseline characteristics of patients.

Characteristics	Nephrology Care Status			*p*-Value
	Late (n = 29,615, 38.61%)	Early Infrequent (n = 17,411, 22.70%)	Early Frequent (n = 29,676, 38.69%)	
**Age, mean (SD)**	61.80 (15.23)	63.19 (14.04)	63.45 (13.30)	<0.0001
Age, n (%)				
<65	16,115 (54.41)	8792 (50.50)	14,870 (50.11)	<0.0001
65+	13,500 (45.59)	8619 (49.50)	14,806 (49.89)	
Sex, n (%)				
Male	15,586 (52.63)	8660 (49.74)	13,764 (46.38)	<0.0001
Female	14,029 (47.37)	8751 (50.26)	15,912 (53.62)	
Economic status, n (%)				
Low	14,550 (49.13)	8257 (47.42)	13,260 (44.68)	<0.0001
High	15,065 (50.87)	9154 (52.58)	16,416 (55.32)	
Dialysis modality, n (%)				
Hemodialysis	27,109 (91.54)	15,956 (91.64)	25,799 (86.94)	<0.0001
Peritoneal dialysis	2506 (8.46)	1455 (8.36)	3877 (13.06)	
ESRD initiation year, n (%)				
1999	2375 (8.02)	1256 (7.21)	1307 (4.40)	<0.0001
2000	2369 (8.00)	1239 (7.12)	1556 (5.24)	
2001	2371 (8.01)	1328 (7.63)	1892 (6.38)	
2002	2446 (8.26)	1363 (7.83)	2103 (7.09)	
2003	2383 (8.05)	1536 (8.82)	2320 (7.82)	
2004	2508 (8.47)	1569 (9.01)	2199 (7.41)	
2005	2528 (8.54)	1650 (9.48)	2641 (8.90)	
2006	2444 (8.25)	1557 (8.94)	2424 (8.17)	
2007	2604 (8.79)	1480 (8.50)	2881 (9.71)	
2008	2597 (8.77)	1474 (8.47)	3115 (10.50)	
2009	2415 (8.15)	1489 (8.55)	3445 (11.61)	
2010	2575 (8.69)	1470 (8.44)	3793 (12.78)	
Prior hospitalization, n (%)				
Yes	27,551 (93.03)	16,208 (93.09)	25,677 (86.52)	<0.0001
No	2064 (6.97)	1203 (6.91)	3999 (13.48)	
Prior physician visits, mean (SD)	28.77 (20.59)	35.22 (21.90)	39.25 (19.82)	<0.0001
Prior physician visits, n (%)				
Low (0–23)	13,774 (46.51)	5765 (33.11)	5909 (19.91)	<0.0001
Intermediate (24–39)	8775 (29.63)	5717 (32.84)	11,929 (40.20)	
High (40+)	7066 (23.86)	5929 (34.05)	11,838 (39.89)	
Deyo’s Charlson Comorbidity Index, mean (SD)	4.16 (2.26)	4.80 (2.30)	4.50 (2.13)	<0.0001
Deyo’s Charlson Comorbidity Index, n (%)				
<4	13,119 (44.30)	5731 (32.92)	11,397 (38.40)	<0.0001
4+	16,496 (55.70)	11,680 (67.08)	18,279 (61.60)	
Comorbidity, n (%)				
Arrhythmia	2114 (7.14)	1447 (8.31)	1931 (6.51)	<0.0001
Cancer	2199 (7.43)	1387 (7.97)	2260 (7.62)	0.10
Cerebral vascular disease	3794 (12.81)	2288 (13.14)	2753 (9.28)	<0.0001
Chronic liver disease	2918 (9.85)	1939 (11.14)	2870 (9.67)	<0.0001
Chronic obstructive pulmonary disease	2921 (9.86)	1921 (11.03)	2346 (7.91)	<0.0001
Coronary artery disease	2879 (9.72)	2081 (11.95)	2522 (8.50)	<0.0001
Dementia	1102 (3.72)	741 (4.26)	876 (2.95)	<0.0001
Diabetes	15,121 (51.06)	9972 (57.27)	15,647 (52.73)	<0.0001
Dyslipidemia	4847 (16.37)	3446 (19.79)	6495 (21.89)	<0.0001
Gout	3201 (10.81)	2508 (14.40)	4981 (16.78)	<0.0001
Heart failure	13,508 (45.61)	9174 (52.69)	13,140 (44.28)	<0.0001
Hypertension	19,901 (67.20)	13,226 (75.96)	22,538 (75.95)	<0.0001
Osteoporosis	1406 (4.75)	1165 (6.69)	1841 (6.20)	<0.0001
Peptic ulcer disease	4462 (15.07)	3208 (18.43)	4879 (16.44)	<0.0001
Peripheral vascular disease	833 (2.81)	492 (2.83)	724 (2.44)	0.007
Psychiatric disorder	1524 (5.15)	1197 (6.87)	1936 (6.52)	<0.0001
Vascular heart disease	1940 (6.55)	1260 (7.24)	1565 (5.27)	<0.0001

**Table 2:** Characteristics for ESRD patients and 1-year post-dialysis ED visits.

		All Patients (n = 76,702)	Nephrology Care Status		
			Late (n = 29,615, 38.61%)	Early Infrequent (n = 17,411, 22.70%)	Early Frequent (n = 29,676, 38.69%)
All-cause	ED visits No. (%) of patients	44,714 (58.30)	16,852 (56.90)	10,462 (60.09)	17,400 (58.63)
	Mean No. of ED visits per patient-year	1.73	1.70	1.89	1.66
	ED visits ≥ 3 times, N (%)	16,651 (21.71)	6078 (20.52)	4172 (23.96)	6401 (21.57)
Infection-related	ED visits No. (%) of patients	13,353 (17.41)	5314 (17.94)	3243 (18.63)	4796 (16.16)
	Mean No. of ED visits per patient-year	0.28	0.30	0.30	0.25
	ED visits ≥ 3 times, N (%)	1405 (1.83)	580 (1.96)	334 (1.92)	491 (1.65)
Avoidable	ED visits No. (%) of patients	5352 (6.98)	2138 (7.22)	1368 (7.86)	1846 (6.22)
	Mean No. of ED visits per patient-year	0.09	0.10	0.11	0.08
	ED visits ≥ 3 times, N (%)	204 (0.27)	95 (0.32)	55 (0.32)	54 (0.18)

**Table 3:** Adjusted risk for 1-year EDs based on predialysis nephrology care status.

Outcome and Predialysis Nephrology Care Status ^a^		COX Proportional Model			Recurrent Event Analysis		
		HR	95% CI		RR	95% CI	
All-cause ED visit	Early frequent	0.92	0.90	0.94	0.94	0.93	0.96
	Early infrequent	1.02	0.99	1.04	1.01	0.99	1.02
Infection-related ED visit	Early frequent	0.76	0.73	0.79	0.84	0.82	0.87
	Early infrequent	0.94	0.90	0.98	0.96	0.93	0.99
Avoidable ED visit	Early frequent	0.76	0.71	0.81	0.79	0.75	0.84
	Early infrequent	0.95	0.89	1.02	0.97	0.91	1.03

Notes: Adjusted for ESRD initiation year, age, sex, economic status, dialysis modality, prior hospitalization, prior physician visits, and comorbid conditions (arrhythmia, cancer, cerebral vascular disease, chronic liver disease, chronic obstructive pulmonary disease, coronary artery disease, dementia, diabetes, dyslipidemia, gout, heart failure, hypertension, osteoporosis, peptic ulcer disease, peripheral vascular disease, psychiatric disorder, vascular heart disease). Abbreviations: ED, emergency department; CI, confidence interval; HR, hazard ratio; RR, relative risk. ^a^ Reference group is late nephrology care.

## Appendix A

**Table A1:** Baseline characteristics of patients with and without ED visit.

Characteristics	All-Cause ED Visit		*p*-Value	Infection-Related ED Visit		*p*-Value	Potentially Avoidable ED Visit		*p*-Value
	No (n = 31,988, 41.70%)	Yes (n = 44,714, 58.30%)		No (n = 63349, 82.59%)	Yes (n = 13353, 17.41%)		No (n = 71,350, 93.02%)	Yes (n = 5352, 6.98%)	
Age, mean (SD)	60.90 (14.39)	64.09 (14.01)	<0.0001	61.86 (14.21)	67.03 (13.67)	<0.0001	62.54 (14.29)	65.58 (13.44)	<0.0001
Age, n (%)									
<65	18,418 (57.58)	21,359 (47.77)	<0.0001	34,615 (54.64)	5162 (38.66)	<0.0001	37,412 (52.43)	2365 (44.19)	<0.0001
65+	13,570 (42.42)	23,355 (52.23)		28,734 (45.36)	8191 (61.34)		33,938 (47.57)	2987 (55.81)	
Sex, n (%)									
Male	16,017 (50.07)	21,993 (49.19)	0.02	31,562 (49.82)	6448 (48.29)	0.001	35,438 (49.67)	2572 (48.06)	0.02
Female	15,971 (49.93)	22,721 (50.81)		31,787 (50.18)	6905 (51.71)		35,912 (50.33)	2780 (51.94)	
Economic status, n (%)									
Low	14,568 (45.54)	21,499 (48.08)	<0.0001	29,558 (46.66)	6509 (48.75)	<0.0001	33,385 (46.79)	2682 (50.11)	<0.0001
High	17,420 (54.46)	23,215 (51.92)		33,791 (53.34)	6844 (51.25)		37,965 (53.21)	2670 (49.89)	
Dialysis modality, n (%)									
Hemodialysis	28,240 (88.28)	40,624 (90.85)	<0.0001	56,736 (89.56)	12,128 (90.83)	<0.0001	63,886 (89.54)	4978 (93.01)	<0.0001
Peritoneal dialysis	3748 (11.72)	4090 (9.15)		6613 (10.44)	1225 (9.17)		7464(10.46)	374 (6.99)	
ESRD initiation year, n (%)									
1999	3001 (9.38)	1937 (4.33)	<0.0001	4653 (7.35)	285 (2.13)	<0.0001	4768 (6.68)	170 (3.18)	<0.0001
2000	3413 (10.67)	1751 (3.92)		4776 (7.54)	388 (2.91)		4952 (6.94)	212 (3.96)	
2001	2242 (7.01)	3349 (7.49)		4807 (7.59)	784 (5.87)		5163 (7.24)	428 (8.00)	
2002	2277 (7.12)	3635 (8.13)		5028 (7.94)	884 (6.62)		5468 (7.66)	444 (8.30)	
2003	3337 (10.43)	2902 (6.49)		5459 (8.62)	780 (5.84)		5877 (8.24)	362 (6.76)	
2004	3608 (11.28)	2668 (5.97)		5473 (8.64)	803 (6.01)		5957 (8.35)	319 (5.96)	
2005	2424 (7.58)	4395 (9.83)		5571 (8.79)	1248 (9.35)		6259 (8.77)	560 (10.46)	
2006	2250 (7.03)	4175 (9.34)		5079 (8.02)	1346 (10.08)		5910 (8.28)	515 (9.62)	
2007	2363 (7.39)	4602 (10.29)		5467 (8.63)	1498 (11.22)		6425 (9.00)	540 (10.09)	
2008	2280 (7.13)	4906 (10.97)		5510 (8.70)	1676 (12.55)		6579 (9.22)	607 (11.34)	
2009	2368 (7.40)	4981 (11.14)		5608 (8.85)	1741 (13.04)		6737 (9.44)	612 (11.43)	
2010	2425 (7.58)	5413 (12.11)		5918 (9.34)	1920 (14.38)		7255 (10.17)	583 (10.89)	
Prior hospitalization, n (%)									
Yes	28,088 (87.81)	41,348 (92.47)	<0.0001	56,763 (89.60)	12,673 (94.91)	<0.0001	64,360 (90.20)	5076 (94.84)	<0.0001
No	3900 (12.19)	3366 (7.53)		6586(10.40)	680 (5.09)		6990 (9.80)	276 (5.16)	
Prior physician visits, mean (SD)	32.35 (20.21)	35.67 (21.64)	<0.0001	33.77 (20.79)	36.71 (22.46)	<0.0001	33.97 (20.96)	38.43 (22.75)	<0.0001
Prior physician visits, n (%)									
Low (0–23)	11,726 (36.66)	13, 722 (30.69)	<0.0001	21,545 (34.01)	3903 (29.23)	<0.0001	24,069 (33.73)	1379 (25.77)	<0.0001
Intermediate (24–39)	11,011 (34.42)	15,410 (34.46)		21,864 (34.51)	4557 (34.13)		24,594 (34.47)	1827 (34.14)	
High (40+)	9251 (28.92)	15,582 (34.85)		19,940 (31.48)	4893 (36.64)		22,687 (31.80)	2146 (40.10)	
CCI, mean (SD)	4.08 (2.13)	4.69 (2.27)	<0.0001	4.33 (2.19)	4.96 (2.36)	<0.0001	4.38 (2.22)	5.22 (2.26)	<0.0001
CCI, n (%)									
<4	14,923 (46.65)	15,324 (34.27)	<0.0001	26,275 (41.48)	3972 (29.75)	<0.0001	28,957 (40.58)	1290 (24.10)	<0.0001
4+	17,065 (53.35)	29,390 (65.73)		37,074 (58.52)	9381 (70.25)		42,393 (59.42)	4062 (75.90)	
Comorbidity, n (%)									
Arrhythmia	2005 (6.27)	3487 (7.80)	<0.0001	4297 (6.78)	1195 (8.95)	<0.0001	4996 (7.00)	496 (9.27)	<0.0001
Cancer	2047 (6.40)	3799 (8.50)	<0.0001	4533 (7.16)	1313 (9.83)	<0.0001	5468 (7.66)	378 (7.06)	0.11
Cerebral vascular disease	3168 (9.90)	5667 (12.67)	<0.0001	6626 (10.46)	2209 (16.54)	<0.0001	7964 (11.16)	871 (16.27)	<0.0001
Chronic liver disease	2896 (9.05)	4831 (10.80)	<0.0001	6144 (9.70)	1583 (11.86)	<0.0001	7174 (10.05)	553 (10.33)	0.51
COPD	2792 (8.73)	4396 (9.83)	<0.0001	5492 (8.67)	1696 (12.70)	<0.0001	6500 (9.11)	688 (12.86)	<0.0001
Coronary artery disease	2466 (7.71)	5016 (11.22)	<0.0001	5769 (9.11)	1713 (12.83)	<0.0001	6714 (9.41)	768 (14.35)	<0.0001
Dementia	918 (2.87)	1801 (4.03)	<0.0001	1818 (2.87)	901 (6.75)	<0.0001	2428 (3.40)	291 (5.44)	<0.0001
Diabetes	14,830 (46.36)	25,910 (57.95)	<0.0001	32,390 (51.13)	8350 (62.53)	<0.0001	36,844 (51.64)	3896 (72.80)	<0.0001
Dyslipidemia	5470 (17.10)	9318 (20.84)	<0.0001	12,106 (19.11)	2682 (20.09)	0.009	13,537 (18.97)	1251 (23.37)	<0.0001
Gout	4233 (13.23)	6457 (14.44)	<0.0001	8751 (13.81)	1939 (14.52)	0.03	10,044 (14.08)	646 (12.07)	<0.0001
Heart failure	13,753 (42.99)	22,069 (49.36)	<0.0001	28,914 (45.64)	6908 (51.73)	<0.0001	32,685 (45.81)	3137 (58.61)	<0.0001
Hypertension	22,536 (70.45)	33,129 (74.09)	<0.0001	45758 (72.23)	9907 (74.19)	<0.0001	51,457 (72.12)	4208 (78.62)	<0.0001
Osteoporosis	1653 (5.17)	2759 (6.17)	<0.0001	3424 (5.40)	988 (7.40)	<0.0001	4034 (5.65)	378 (7.06)	<0.0001
Peptic ulcer disease	4591 (14.35)	7958 (17.80)	<0.0001	9882 (15.60)	2667 (19.97)	<0.0001	11,517 (16.14)	1032 (19.28)	<0.0001
Peripheral vascular disease	739 (2.31)	1310 (2.93)	<0.0001	1599 (2.52)	450 (3.37)	<0.0001	1866 (2.62)	183 (3.42)	0.0004
Psychiatric disorder	1578 (4.93)	3079 (6.89)	<0.0001	3603 (5.69)	1054 (7.89)	<0.0001	4191 (5.87)	466 (8.71)	<0.0001
Vascular heart disease	1719 (5.37)	3046 (6.81)	<0.0001	3789 (5.98)	976 (7.31)	<0.0001	4315 (6.05)	450 (8.41)	<0.0001

Abbreviations: CCI, Deyo’s Charlson Comorbidity Index; COPD, Chronic obstructive pulmonary disease.

## References

[1] Wetmore J.B., Collins A.J. (2016). Global challenges posed by the growth of end-stage renal disease. Ren. Replace. Ther..

[2] Saran R., Robinson B., Abbott K.C., Agodoa L.Y., Albertus P., Ayanian J., Balkrishnan R., Bragg-Gresham J., Cao J., Chen J.L. (2017). US renal data system 2016 annual data report: Epidemiology of kidney disease in the United States. Am. J. Kidney Dis..

[3] Philipneri M.D., Rocca Rey L.A., Schnitzler M.A., Abbott K.C., Brennan D.C., Takemoto S.K., Buchanan P.M., Burroughs T.E., Willoughby L.M., Lentine K.L. (2008). Delivery patterns of recommended chronic kidney disease care in clinical practice: Administrative claims-based analysis and systematic literature review. Clin. Exp. Nephrol..

[4] Tisher C.C., Bastl C.P., Bistrian B.R., Chesney R., Coggins C., Diener-West M., Fanestil D.D., Grantham J., Kunau R., Luke R.G. (1994). Morbidity and mortality of renal dialysis: An NIH consensus conference statement. Ann. Intern. Med..

[5] Nakamura S., Nakata H., Yoshihara F., Kamide K., Horio T., Nakahama H., Kawano Y. (2007). Effect of early nephrology referral on the initiation of hemodialysis and survival in patients with chronic kidney disease and cardiovascular diseases. Circ. J..

[6] Smart N.A., Dieberg G., Ladhani M., Titus T. (2014). Early referral to specialist nephrology services for preventing the progression to end-stage kidney disease. Cochrane Database Syst. Rev..

[7] Lee J., Lee J.P., Park J.I., Hwang J.H., Jang H.M., Choi J.Y., Kim Y.L., Yang C.W., Kang S.W., Kim N.H. (2014). Early Nephrology Referral Reduces the Economic Costs among Patients Who Start Renal Replacement Therapy: A Prospective Cohort Study in Korea. PLoS ONE.

[8] Yang J.Y., Huang J.W., Chen L., Chen Y.Y., Pai M.F., Tung K.T., Peng Y.S., Hung K.Y. (2017). Frequency of Early Predialysis Nephrology Care and Postdialysis Cardiovascular Events. Am. J. Kidney Dis..

[9] See E.J., Johnson D.W., Hawley C.M., Pascoe E.M., Badve S.V., Boudville N., Clayton P.A., Sud K., Polkinghorne K.R., Borlace M. (2018). Risk Predictors and Causes of Technique Failure Within the First Year of Peritoneal Dialysis: An Australia and New Zealand Dialysis and Transplant Registry (ANZDATA) Study. Am. J. Kidney Dis..

[10] Winkelmayer W.C., Owen W.F., Levin R., Avorn J. (2003). A propensity analysis of late versus early nephrologist referral and mortality on dialysis. J. Am. Soc. Nephrol..

[11] Kumar S., Jeganathan J.A. (2012). Timing of nephrology referral: Influence on mortality and morbidity in chronic kidney disease. Nephrourol. Mon..

[12] Smart N.A., Titus T.T. (2011). Outcomes of Early versus Late Nephrology Referral in Chronic Kidney Disease: A Systematic Review. Am. J. Med..

[13] Dogan E., Erkoc R., Sayarlioglu H., Durmus A., Topal C. (2005). Effects of late referral to a nephrologist in patients with chronic renal failure. Nephrology.

[14] Roubicek C., Brunet P., Huiart L., Thirion X., Leonetti F., Dussol B., Jaber K., Andrieu D., Ramananarivo P., Berland Y. (2000). Timing of nephrology referral: Influence on mortality and morbidity. Am. J. Kidney Dis..

[15] Black C., Sharma P., Scotland G., McCullough K., McGurn D., Robertson L., Fluck N., MacLeod A., McNamee P., Prescott G. (2010). Early referral strategies for management of people with markers of renal disease: A systematic review of the evidence of clinical effectiveness, cost-effectiveness and economic analysis. Health Technol. Assess..

[16] Astor B.C., Eustace J., Powe N., Klag M.J., Sadler J.H., Fink N.E., Coresh J. (2001). Timing of nephrologist referral and arteriovenous access use: The CHOICE study. Am. J. Kidney Dis..

[17] Stack A.G. (2002). Determinants of modality selection among incident US dialysis patients: Results from a national study. J. Am. Soc. Nephrol..

[18] Miller J.B., Brauer E., Rao H., Wickenheiser K., Dev S., Omino R., Stokes-Buzzelli S. (2013). The most frequent ED patients carry insurance and a significant burden of disease. Am. J. Emerg. Med..

[19] Morris J.N., Howard E.P., Steel K., Schreiber R., Fries B.E., Lipsitz L.A., Goldman B. (2014). Predicting risk of hospital and emergency department use for home care elderly persons through a secondary analysis of cross-national data. BMC Health Serv. Res..

[20] Lin Y.C., Hsu H.K., Lai T.S., Chiang W.C., Lin S.L., Chen Y.M., Chen C.C., Chu T.S. (2019). Emergency department utilization and resuscitation rate among patients receiving maintenance hemodialysis. J. Med. Assoc..

[21] Komenda P., Tangri N., Klajncar E., Eng A., Di Nella M., Hiebert B., Strome T., de Faria L.R., Zacharias J.M., Verrelli M. (2018). Patterns of emergency department utilization by patients on chronic dialysis: A population-based study. PLoS ONE.

[22] Lovasik B.P., Zhang R., Hockenberry J.M., Schrager J.D., Pastan S.O., Mohan S., Patzer R.E. (2016). Emergency department use and hospital admissions among patients with end-stage renal disease in the United States. JAMA Intern. Med..

[23] Dalrymple L.S., Johansen K.L., Chertow G.M., Cheng S.-C., Grimes B., Gold E.B., Kaysen G.A. (2010). Infection-related hospitalizations in older patients with ESRD. Am. J. Kidney Dis..

[24] Agency for Healthcare Research and Quality (AHRQ) Prevention Quality Indicators Technical Specifications Updates—Version 6.0 (ICD-9), October 2016. https://www.qualityindicators.ahrq.gov/Modules/PQI_TechSpec_ICD09_v60.aspx.

[25] Lin D.Y., Wei L.J., Yang I., Ying Z. (2000). Semiparametric regression for the mean and rate functions of recurrent events. J. R. Stat. Soc. Ser. B Stat. Methodol..

[26] Kim D.H., Kim M., Kim H., Kim Y.L., Kang S.W., Yang C.W., Kim N.H., Kim Y.S., Lee J.P. (2013). Early Referral to a Nephrologist Improved Patient Survival: Prospective Cohort Study for End-Stage Renal Disease in Korea. PLoS ONE.

[27] Schmidt R.J., Domico J.R., Sorkin M.I., Hobbs G. (1998). Early referral and its impact on emergent first dialyses, health care costs, and outcome. Am. J. Kidney Dis..

[28] Hoen B., Paul-Dauphin A., Hestin D., Kessler M. (1998). EPIBACDIAL: A multicenter prospective study of risk factors for bacteremia in chronic hemodialysis patients. J. Am. Soc. Nephrol..

[29] Lemaire X., Morena M., Leray-Moragués H., Henriet-Viprey D., Chenine L., Defez-Fougeron C., Canaud B. (2009). Analysis of risk factors for catheter-related bacteremia in 2000 permanent dual catheters for hemodialysis. Blood Purif..

[30] Farag A., Garg A.X., Li L., Jain A.K. (2014). Dosing errors in prescribed antibiotics for older persons with CKD: A retrospective time series analysis. Am. J. Kidney Dis..

[31] Gallieni M., Cancarini G. (2015). Drugs in the elderly with chronic kidney disease: Beware of potentially inappropriate medications. Nephrol. Dial. Transpl..

[32] Budnitz D.S., Shehab N., Kegler S.R., Richards C.L. (2007). Medication use leading to emergency department visits for adverse drug events in older adults. Ann. Intern. Med..

[33] Zhu J.X., Nash D.M., McArthur E., Farag A., Garg A.X., Jain A.K. (2018). Nephrology comanagement and the quality of antibiotic prescribing in primary care for patients with chronic kidney disease: A retrospective cross-sectional study. Nephrol. Dial. Transpl..

[34] Kazmi W.H., Obrador G.T., Khan S.S., Pereira B.J., Kausz A.T. (2004). Late nephrology referral and mortality among patients with end-stage renal disease: A propensity score analysis. Nephrol. Dial. Transpl..

[35] Gallego E., López A., Lorenzo I., López E., Llamas F., Illescas M.L., Andrés E., Serrano A., Olivas E., Gómez Roldán C. (2003). Influence of early or late referral to nephrologist over morbidity and mortality in hemodialysis. Nefrologia.

[36] Jungers P., Joly D., Nguyen-Khoa T., Mothu N., Bassilios N., Grünfeld J.P. (2006). Continued late referral of patients with chronic kidney disease. Presse Med..

[37] Di Napoli A., Valle S., d’Adamo G., Pezzotti P., Chicca S., Pignocco M., Spinelli C., Di Giulio S., Di Lallo D., Predialysis Study Group of Lazio (2010). Survey of determinants and effects of timing of referral to a nephrologist: The patient’s point of view. J. Nephrol..

[38] Quaglia M., Canavese C., Stratta P. (2011). Early Nephrology Referral: How Early Is Early Enough?. Arch. Intern. Med..

